# The lncRNA MIR4435-2HG is upregulated in hepatocellular carcinoma and promotes cancer cell proliferation by upregulating miRNA-487a

**DOI:** 10.1186/s11658-019-0148-y

**Published:** 2019-04-04

**Authors:** Qinglei Kong, Caiqian Liang, Yi Jin, Yuhang Pan, Dayue Tong, Qingcong Kong, Jing Zhou

**Affiliations:** 1 Department of Emergency Medicine, The Third Affiliated Hospital, Sun Yat-Sen University, Guangzhou City, Guangdong Province 510630 People’s Republic of China; 2 Department of Pathology, Guangdong Provincial Key Laboratory of Liver Disease Research, The Third Affiliated Hospital, Sun Yat-Sen University, No. 600 Tianhe Road, Guangzhou City, Guangdong Province 510630 People’s Republic of China; 3 Department of Forensic Medicine, ZhongShan Medical School, Sun Yat-Sen University, Guangzhou City, Guangdong Province 510080 People’s Republic of China; 4 Department of Radiology, The Third Affiliated Hospital, Sun Yat-Sen University, No. 600 Tianhe Road, Guangzhou City, Guangdong Province 510630 People’s Republic of China

**Keywords:** Hepatocellular carcinoma, lncRNA MIR4435-2HG, miRNA-487a, Proliferation

## Abstract

**Background:**

Given the high mortality rate and unclear pathogenesis for liver cancer, investigation of its molecular mechanisms is essential. We focused on the long non-coding RNA (lncRNA) MIR4435-2HG, which was recently reported to be oncogenic in lung cancer and the microRNA miRNA-487a, which has been reported to be oncogenic in hepatocellular carcinoma (HCC). Our aim was to determine if the former has a role in HCC, and to further validate the role of the latter.

**Methods:**

Samples from 64 patients with HCC were taken at The Third Affiliated Hospital of Sun Yat-Sen University. Cell transfection and PCR were applied.

**Results:**

We found that MIR4435-2HG and miRNA-487a were upregulated in tumor tissues compared to adjacent healthy tissues from HCC patients. The expression of MIR4435-2HG was significantly affected by tumor size but not by tumor metastasis. Correlation analysis showed that MIR4435-2HG and miRNA-487a were positively correlated in both the tumor tissues and adjacent healthy tissues from HCC patients. Overexpression of MIR4435-2HG led to upregulation of miRNA-487a in the cells of HCC cell lines, while overexpression of miRNA-487a did not significantly affect MIR4435-2HG. Overexpression of MIR4435-2HG and miRNA-487a promoted the proliferation of cells of HCC cell lines, and miRNA-487a knockdown partially attenuated the enhancing effects of MIR4435-2HG overexpression on cancer cell proliferation.

**Conclusion:**

MIR4435-2HG is upregulated in HCC and promotes cancer cell proliferation possibly by upregulating miRNA-487a.

## Background

With more than 45,000 deaths annually worldwide, liver cancer is the second leading cause of mortality in cancer patients [1]. Thanks to the popularization of liver disease screening programs and the improved characterization of risk factors for liver cancer, its incidence has not increased in the last few decades in some regions, including the US [2–4]. However, its mortality rate has increased [2]. The unclear pathogenesis of liver cancer is the main cause of failure in clinical treatment [5]. In-depth investigations into the molecular mechanism of the occurrence and development of liver cancer may guide treatment.

The human genome encodes both protein-coding messenger RNAs (mRNAs) and non-coding RNAs (ncRNAs). Although ncRNAs are critical determinants in cancer biology, the function of most remains unknown. Unlike mRNAs, ncRNAs directly participate in physiological and pathological processes [6, 7].

Long non-coding RNAs (lncRNAs) and microRNAs (miRNAs) are two major subgroups of ncRNAs. Both have been proven to be critical players in cancer biology [8, 9]. The lncRNA MIR4435-2HG was recently reported to be oncogenic in lung cancer [10]. The microRNA miRNA-487a has been reported to be oncogenic in hepatocellular carcinoma (HCC), which is a major subtype of liver cancer [11]. During the development of HCC, overexpression of miRNA-487a has been proven to be responsible for the increased proliferation and migration of cancer cells [11]. In this study, we showed that MIR4435-2HG was upregulated in HCC and promoted cancer cell proliferation, possibly by upregulating miRNA-487a.

## Methods

### Human subjects

Our study included 64 patients with HCC who were admitted to Third Affiliated Hospital of Sun Yat-Sen University between May 2016 and July 2018. All patients were diagnosed using pathological examinations. Biopsies of tumor tissue and adjacent healthy tissue (within 2 cm of the tumors) were obtained from each patient. All tissues were assessed by 3 experienced pathologists. Inclusion criteria were: 1) first-time diagnosis; 2) complete medical record; 3) willingness to join the study. Exclusion criteria were: 1) presence of other diseases, such as liver and chronic diseases; 2) treatment for HCC before admission; 3) refusal to provide liver biopsies. There were 39 males and 25 females. The age range was 35 to 66 years, with a mean age of 46.1 ± 4.9 years. There were 10 cases of stage I, 12 cases of stage II, 11 cases of stage III and 31 cases of stage IV. This study passed the review of the Ethics Committee of The Third Affiliated Hospital of Sun Yat-Sen University. All participants signed informed consent.

### Quantitative RT-PCR

To detect the expression of MIR4435-2HG, total RNA was extracted using an MPure Total RNA Extraction Kit (117,022,160, MP Biomedicals). Reverse transcription was performed using a gb Reverse Transcription Kit (Generi Biotech). PCR systems were prepared using a Luna Universal One-Step RT-qPCR Kit (NEB). To detect the expression of miRNA-487a, an miRNeasy Mini Kit (QIAGEN) was used to extract miRNA. A TaqMan MicroRNA Reverse Transcription Kit (Thermo Fisher Scientific) was used to perform reverse transcription and a miScript SYBR Green PCR Kit (QIAGEN) was used to prepare PCR systems. Primers of MIR4435-2HG, miRNA-487a and the endogenous controls β-actin and U6 were synthesized at Sangon. Using the 2^-ΔΔCT^ method, the expression of MIR4435-2HG was normalized to β-actin and the expression of miRNA-487a was normalized to U6.

### Cell lines and cell transfection

Our study included two HCC cell lines: SNU-398 and SNU-182. Cells of these two lines were bought from the American Type Culture Collection (ATCC) and cultivated under the conditions recommended by the ATCC. Vectors expressing MIR4435-2HG, empty vectors, the hsa-miR-487a mimic (AAUCAUACAGGGACAUCCAGUU) and the negative control miRNA were purchased from Sangon. The hsa-miR-487a inhibitor and miRNA inhibitor negative control were purchased from ABM. Lipofectamine 3000 reagent (Thermo Fisher Scientific) was used to perform all cell transfections, with all operations performed according to the manufacturer’s instructions. The treatment regime was 10 mM vectors and 45 mM miRNA. Control cells were only treated with lipofectamine 3000 reagent. Negative control cells were transfected with empty vectors, negative control miRNA or miRNA inhibitor.

### Cell proliferation assay

The expressions of MIR4435-2HG and miR-487a were checked 24 h after transfection. Cell proliferation was determined using a Cell Counting Kit-8 kit (Dojindo Molecular Technologies, Inc.) For cells with 200% overexpression of MIR4435-2HG and miR-487a and cells with miR-487a knockdown, where its expression was 50%. Briefly, cells were collected to prepare cell suspensions. The cell density was adjusted to 3 × 10^4^ cells/ml and was transferred to a 96-well plate with 100 μl in each well. Cells were cultivated in an incubator (5% CO_2_, 37 °C), and 10 μl CCK-8 was added to each well after 24, 48, 72 and 96 h. OD values at 450 nm were measured to represent cell proliferation.

### Statistical analysis

All experiments in this study were performed in triplicate. Data are expressed as means ± SD. Comparisons of MIR4435-2HG and miRNA-487a expression between tumor and healthy tissues were performed using the paired t test. Comparisons among 3 groups were performed using one-way ANOVA and Tukey test. Correlations between expression levels of MIR4435-2HG and miRNA-487a were performed using Pearson’s correlation coefficient. Differences with *p* < 0.05 were statistically significant.

## Results

### MIR4435-2HG and miRNA-487a were upregulated in tumor tissues of HCC patients

**Fig. 1:**
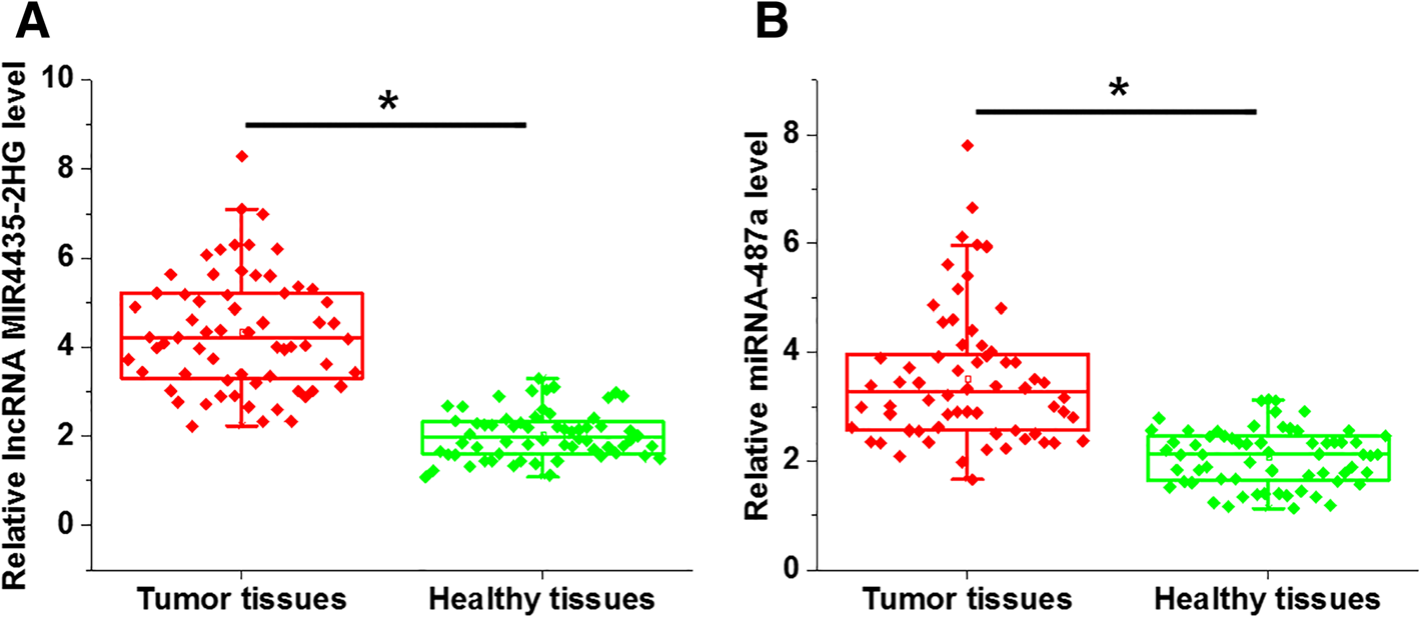
MIR4435-2HG and miRNA-487a were upregulated in tumor tissues of HCC patients. Quantitative RT-PCR results showed thatthe expression levels of MIR4435-2HG (**a**) and miRNA-487a (**b**) were significantly higher in tumor tissues, than in adjacent tissues (*p* < 0.05)

The expression of MIR4435-2HG and miRNA-487a in tumor tissues and adjacent healthy tissues of patients with HCC were determined using quantitative RT-PCR. The expression levels of MIR4435-2HG (Fig. 1a, *p* = 0.012) and miRNA-487a (Fig. 1b, *p* = 0.034) were significantly higher in tumor tissues than in their adjacent tissues.

### Expression of MIR4435-2HG was significantly affected by tumor size but not by tumor metastasis

**Fig. 2:**
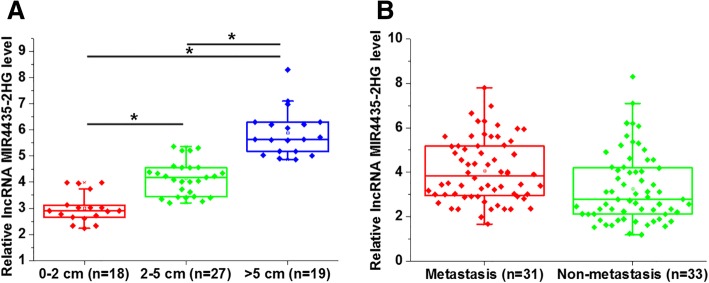
Expression of MIR4435-2HG was significantly affected by tumor size but not by tumor metastasis. The expression level of MIR4435-2HG increased with the increase in tumor diameter (**a**), while expression level of MIR4435-2HG was not significantly different between metastatic and non-metastatic groups (**b**). *p < 0.05

Based on the diameter of primary tumor, patients were divided into 0–2 cm group (*n* = 18), 2–5 cm group (*n* = 27) and > 5 cm group (*n* = 19). As shown in Fig. 2a, expression level of MIR4435-2HG increased with the increase in tumor diameter (*p* = 0.017 or 0.020, left to right). Based on the existence of tumor metastasis, patients were divided into metastatic (*n* = 31) and non-metastatic (*n* = 33) groups. As shown in Fig. 2b, the expression level of MIR4435-2HG was not significantly different between the metastatic and non-metastatic groups (*p* = 0.342).

### MIR4435-2HG and miRNA-487a levels were positively correlated in both tumor tissues and adjacent healthy tissues

**Fig. 3:**
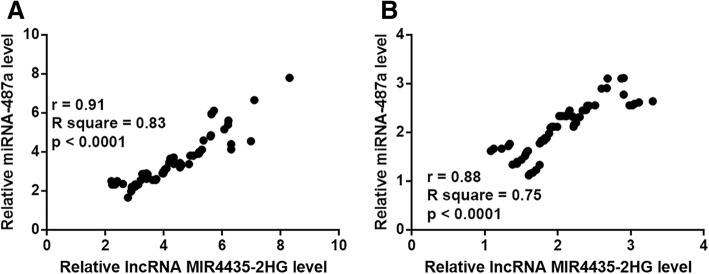
MIR4435-2HG and miRNA-487a expression levels were positively correlated in tumor tissues and adjacent healthy tissues. Pearson’s correlation coefficient showed that MIR4435-2HG and miRNA-487a expression levels were positively correlated in tumor tissues (**a**) and adjacent healthy tissues (**b**) from HCC patients

Correlations between the expression levels of MIR4435-2HG and miRNA-487a were assessed using Pearson’s correlation coefficient. The results showed that MIR4435-2HG and miRNA-487a were positively correlated in both the tumor tissues (Fig. 3a) and adjacent healthy tissues (Fig. 3b) of HCC patients.

### Overexpression of MIR4435-2HG led to upregulation of miRNA-487a in the cells of HCC cell lines

**Fig. 4:**
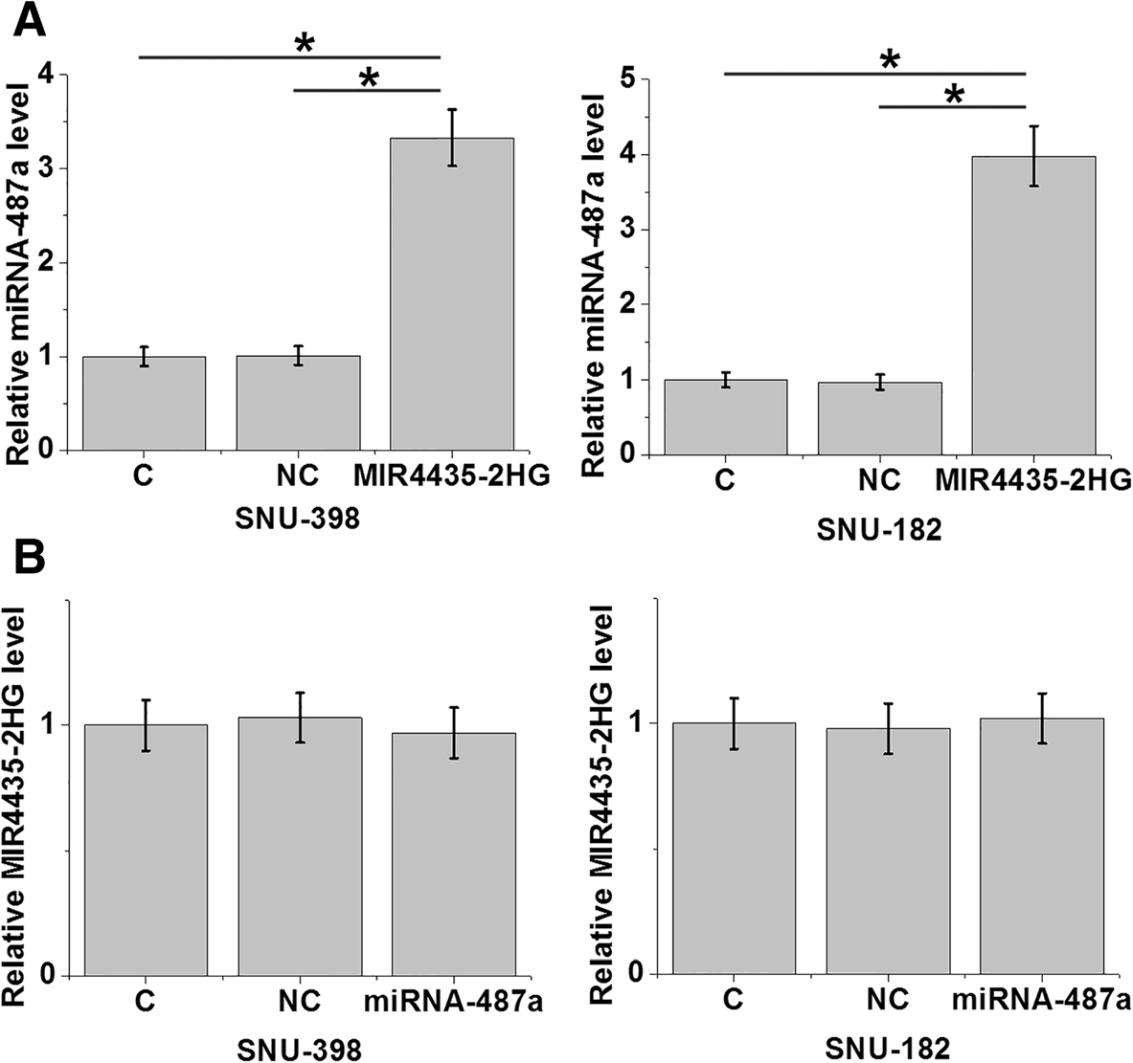
Overexpression of MIR4435-2HG led to upregulation of miRNA-487a in the cells of HCC cell lines. **a** – Overexpression of lncRNA MIR4435-2HG mediated the inhibited expression of miRNA-487a in cells of the HCC cell lines SNU-398 and SNU-182. **b** – overexpression of miRNA-487a did not significantly affect lncRNA MIR4435-2HG. *p < 0.05

Overexpression experiments were performed to investigate the potential interactions between MIR4435-2HG and miRNA-487a in HCC. Compared with the control (C) and negative control (NC) groups, overexpression of MIR4435-2HG led to inhibited expression of miRNA-487a in the cells of the two HCC cell lines SNU-398 (Fig. 4a, *p* < 0.015). and SNU-182 (Fig. 4a, *p* < 0.012). By contrast, overexpression of miRNA-487a did not significantly affect MIR4435-2HG (Fig. 4b, *p* = 0.676).

### MIR4435-2HG regulates HCC cell proliferation through miRNA-487a

**Fig. 5:**
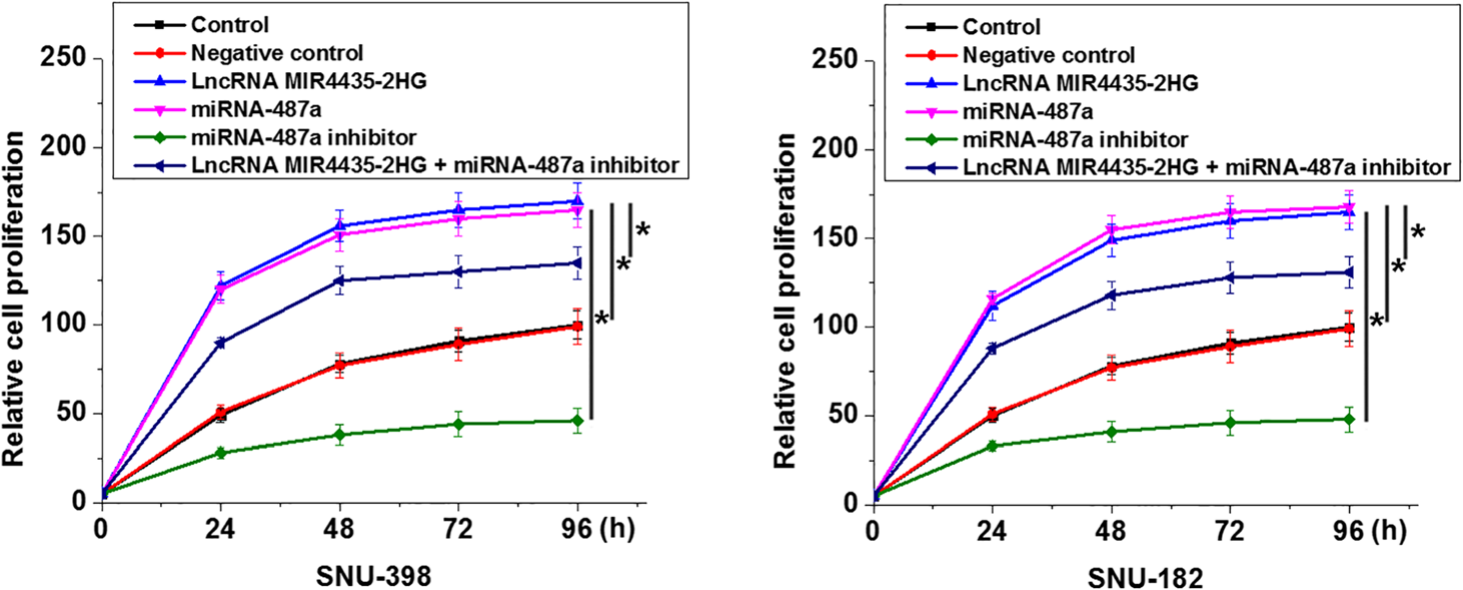
MIR4435-2HG regulates HCC cell proliferation through miRNA-487a. Compared with control (C) and negative control (NC) groups, overexpression of MIR4435-2HG and miRNA-487a led to the promoted proliferation of cells of HCC cell lines. In addition, miRNA-487a knockdown led to the inhibited proliferation of cells of HCC cell lines and partially attenuated the enhancing effects of MIR4435-2HG overexpression on cancer cell proliferation. *p < 0.05

Compared with the control (C) and negative control (NC) cells, overexpression of MIR4435-2HG and miRNA-487a promoted the proliferation of cells of HCC cell lines (Fig. 5, *p* = 0.008 or 0.011, left to right for MIR4435-2HG; *p* = 0.013 or 0.006, left to right for miRNA-487a). In addition, miRNA-487a knockdown partially attenuated the enhancing effects of MIR4435-2HG overexpression on cancer cell proliferation (Fig. 5, *p* = 0.029 or 0.037).

## Discussion

MIR4435-2HG is a recently identified lncRNA with known functionality only in lung cancer. The key findings of this study are that MIR4435-2HG is upregulated in HCC and that this upregulation may promote the proliferation of HCC cell, possibly by upregulating miRNA-487a.

Previous microarray studies have shown that the development and progression of HCC is accompanied by altered expression of lncRNAs and miRNAs [12, 13]. In effect, a growing body of literature has shown that lncRNAs and miRNAs are key players in many types of cancers, including HCC [14]. MIR4435-2HG has been reported to be upregulated in lung cancer [10], indicating its potential role as an oncogenic lncRNA. This is the first report of the upregulation of MIR4435-2HG in tumor tissues of HCC patients. A recent study reported that miRNA-487a is upregulated in HCC [15]. Our study also found upregulation of miRNA-487a in HCC tissues.

The finding in this study that MIR4435-2HG expression in tumor tissues was only affected by tumor size but not by tumor metastasis indicates the involvement of MIR4435-2HG in the growth of HCC. Our in vitro study also proved that overexpression of MIR4435-2HG led to accelerated proliferation of cells of HCC cell lines. Our data also suggest that MIR4435-2HG is an upstream activator of miRNA-487a. Interactions between lncRNAs and miRNAs are involved in the regulation of many biological and pathological processes [16], including the development and progression of HCC [17]. Our study found that upregulation of miRNA-487a by MIR4435-2HG was involved in the regulation of HCC cells. However, whether the interaction between miRNA-487a and MIR4435-2HG is direct or indirect is still unknown. Therefore, more studies are still needed.

## Conclusions

The microRNA miRNA-487a and the lncRNA MIR4435-2HG are both upregulated in HCC. Overexpression of MIR4435-2HG promotes cancer cell proliferation, possibly by upregulating miRNA-487a.

## Abbreviations

HCC: Hepatocellular carcinoma
lncRNAs: Long non-coding RNAs
miRNAs: microRNAs
mRNAs: messenger RNAs
ncRNAs: non-coding RNAs
